# Supporting Spouses’ Caregiving Communication Skills in Dementia Care: A Communication Process Model

**DOI:** 10.1093/geroni/igaf122.2982

**Published:** 2025-12-31

**Authors:** Tyler Nesbit, Emma Bryan, Melissa Armstrong, Carma Bylund, Easton Wollney, Carla Fisher

**Affiliations:** University of Florida, Gainesville, Florida, United States; University of Florida, Gainesville, Florida, United States; University of Florida, Gainesville, Florida, United States; University of Florida, Gainesville, Florida, United States; University of Florida, Gainesville, Florida, United States; University of Florida, Gainesville, Florida, United States

## Abstract

Individuals caring for spouses living with Alzheimer’s disease and Alzheimer’s disease-related dementias (AD/ADRD) report distress tied to caregiver burden. Significant communication skills are required to navigate triadic interactions between caregivers, patients, and clinicians. Caregivers with these skills report less distress, but do not typically receive clinical communication guidance. The evidence-informed cancer-Caregiver Communication Process Model (CCPM) illustrates key communication skills that caregivers enact before, during, and after clinical appointments to attain care-related goals. To adapt CCPM for dementia caregiving, we conducted 15 interviews with AD/ADRD spousal caregivers and thematically analyzed transcripts to confirm and potentially expand the CCPM skills. AD/ADRD caregivers confirmed the importance of the cancer-CCPM skills: they kept lists/notes about what to address before visits, exchanged information or asked questions during visits, and debriefed with their spouse after appointments. Additionally, caregivers identified skills specific to AD/ADRD caregiving, including shift focus/mindset, a communication approach involving multiple skills and implemented before, during, and after appointments to prioritize their spouses’ emotional well-being. For example, before appointments, caregivers primed their spouse to help them manage appointment-related anxiety. During appointments, they emphasized emotionally supportive skills, like reassurances when spouses became confused/upset. After appointments, caregivers incorporated a relational activity/ritual (e.g., getting ice cream) to shift their spouse’s emotional state. Findings support the value of CCPM for caregiver skill development across disease contexts while demonstrating the need for adaptation to provide targeted guidance to caregivers of individuals with dementia. The dementia-CCPM can be implemented in interventions to enhance AD/ADRD caregivers’ clinical communication skills and alleviate distress.

